# TGF-β/Smad signaling during hepatic fibro-carcinogenesis (Review)

**DOI:** 10.3892/ijo.2014.2552

**Published:** 2014-07-22

**Authors:** KATSUNORI YOSHIDA, MIKI MURATA, TAKASHI YAMAGUCHI, KOICHI MATSU ZAKI

**Affiliations:** Department of Gastroenterology and Hepatology, Kansai Medical University, Hirakata, Osaka 573-1010, Japan

**Keywords:** chronic inflammation, fibro-carcinogenesis, hepatitis B, hepatitis C, hepatocellular carcinoma, Smad, TGF-β

## Abstract

After hepatitis virus infection, plasma transforming growth factor (TGF)-β increases in either the acute or chronic inflammatory microenvironment. Although TGF-β is upregulated in patients with hepatocellular carcinoma, it is one of the most potent growth inhibitors for hepatocytes. This cytokine also upregulates extracellular matrix (ECM) production of hepatic stellate cells. Therefore, TGF-β is considered to be the major factor regulating liver carcinogenesis and accelerating liver fibrosis. Smad2 and Smad3 act as the intracellular mediators of TGF-β signal transduction pathway. We have generated numerous antibodies against individual phosphorylation sites in Smad2/3, and identified 3 types of phosphorylated forms (phospho-isoforms): COOH-terminally phosphorylated Smad2/3 (pSmad2C and pSmad3C), linker phosphorylated Smad2/3 (pSmad2L and pSmad3L) and dually phosphorylated Smad2/3 (pSmad2L/C and pSmad3L/C). These Smad phospho-isoforms are categorized into 3 groups: cytostatic pSmad3C signaling, mitogenic pSmad3L signaling and invasive/fibrogenic pSmad2L/C signaling. In this review, we describe differential regulation of TGF-β/Smad signaling after acute or chronic liver injuries. In addition, we consider how chronic inflammation associated with hepatitis virus infection promotes hepatic fibrosis and carcinogenesis (fibro-carcinogenesis), focusing on alteration of Smad phospho-isoform signaling. Finally, we show reversibility of Smad phospho-isoform signaling after therapy against hepatitis virus infection.

## 1. Introduction

Transforming growth factor (TGF)-β is a central regulator in chronic liver disease contributing to fibrogenesis through inflammation (1). Within inflammatory microenvironment, TGF-β is secreted by platelets and Kupffer cells (2). A significant increase in TGF-β expression is observed in the activated hepatic stellate cell (HSC), thus indicating that TGF-β acts as an autocrine positive regulator for ECM production. Responsiveness of ECM production to TGF-β is transient in the process of tissue repair such as liver regeneration after acute liver injury (3,4), thus suggesting that some regulatory mechanisms for the TGF-β signal are present in the activated HSC. In contrast, persistent TGF-β signal associated with the accelerated ECM accumulation is a common finding in human chronic liver diseases of different etiologies (5), indicating that HSC lose their negative regulation for ECM accumulation.

TGF-β inhibits hepatocyte proliferation, but it also promotes hepatocellular carcinoma (HCC). TGF-β has been shown to play both tumor-suppressive and tumor promoting roles (6–8). As disease progresses toward malignancy, HCC gains advantage by selective reduction of the tumor-suppressive activity of TGF-β together with augmentation of TGF-β oncogenic activity (7). In concert with mitogens, TGF-β induces accumulation of extracellular matrix (ECM), while mitogenic signaling antagonizes cytostatic TGF-β function (9,10). Recent studies have emphasized the possibility of the Smad family involvement in the pathogenesis of fibrosis and carcinogenesis (fibro-carcinogenesis).

Because TGF-β is involved in a variety of physiologic processes such as liver regeneration, unraveling the molecular mechanisms of TGF-β signal in a pathologic condition is critical to our understanding of its role in disease and the development of its therapies (1). In this review, we first summarize cell-type specific and context-dependent TGF-β signaling, especially focusing on dynamism of phosphorylated Smad mediators. We next discuss differential regulation of TGF-β/Smad signaling after acute or chronic liver injuries. We then consider how chronic inflammation associated with hepatitis virus infection promotes hepatic fibro-carcinogenesis. Finally, we show reversibility of Smad phospho-isoform signaling after anti-viral therapy.

## 2. TGF-β signaling

### Linker phosphorylation can modify COOH-terminally phosphorylated Smad2/3 signaling

TGF-β pathway involves the receptor-activated Smads (Smad2 and Smad3) through direct serine phosphorylation of COOH termini by TβRI upon TGF-β binding (11). TβRI mediated phosphorylation of Smad2 and Smad3 induces their association with the shared partner Smad4, followed by translocation into the nucleus where these complexes activate transcription of specific genes (12). Smad2 and Smad3 proteins contain a conserved Mad homology (MH)1 domain that binds DNA, and a conserved MH2 domain that binds to receptors, Smad4, and transcription co-activators (11). More divergent linker regions separate the two domains (12). The linker domain undergoes regulatory phosphorylation by Ras/mitogen-activated protein kinase (MAPK) pathways including extracellular signal-regulated kinase (ERK), c-Jun N-terminal kinase (JNK), p38 MAPK, and cyclin-dependent kinase (CDK)-2/4, as well as glycogen synthase kinase 3-β, Ca(2+)-calmodulin-dependent protein kinase II, and G protein-coupled receptor kinase-2 (13–22).

Antibodies (Abs) reactive with structurally-related phosphorylated peptides are emerging as valuable tools for determining phosphorylation sites, and for investigating distinct signals via the phosphorylated domains. To elucidate how linker phosphorylation modulates Smad signaling through COOH-tail phosphorylation, we generated several types of Abs, which selectively react with individual phosphorylated domains in Smad2/3 (23). Domain-specific phospho-Smad2/3 Abs have allowed us to reveal that TβRI and JNK/CDK4 differentially phosphorylate Smad2/3 to create 3 phosphorylated forms (phospho-isoforms): COOH-terminally phosphorylated Smad2/3 (pSmad2C and pSmad3C), linker phosphorylated Smad2/3 (pSmad2L and pSmad3L), and dually phosphorylated Smad2/3 (pSmad2L/C and pSmad3L/C) (23). Except for pSmad2L with cytoplasmic localization (13,24), the other phospho-isoforms are localized to cell nuclei (15,18,19, 22,24–32). Linker phosphorylation can modify COOH-terminally phosphorylated Smad2/3 signaling (13–15,19,20,25). Differential localization of kinases and phosphatases in the cytoplasm or nucleus raises the intriguing possibility of different temporal dynamics for cytoplasmic or nuclear Smad phospho-isoforms, and adds to the repertoire of signaling responses that determine cell-fate decisions (23). Immunohistochemical and immunofluorescence analyses using specific Abs in human tissues can examine clinical significance of context-dependent and cell type-specific signaling mediated by Smad phospho-isoforms, by comparison of tissue/cellular localization of these phospho-isoforms in various pathologic specimens (23).

### Canonical cytostatic TβRI/pSmad3C signaling pathway

**Figure 1:**
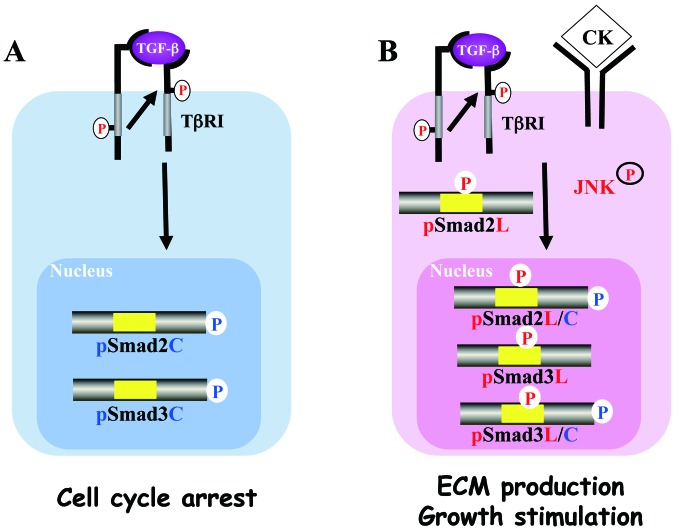
Canonical and non-canonical Smad pathways. TβRI and JNK differentially phosphorylate Smad2/3 to create, 3 phosphorylated forms (phospho-isoforms): COOH-terminally phosphorylated Smad2/3 (pSmad2C and pSmad3C); linker phosphorylated Smad2/3 (pSmad2L and pSmad3L); and dually phosphorylated Smad2/3 (pSmad2L/C and pSmad3L/C). Except for cytoplasmic localization of pSmad2L, the various phospho-isoforms preferentially localize to cell nuclei. (A) Catalytically active TβRI phosphorylates COOH-tail serine residues of Smad2 and Smad3. Both pSmad2C and pSmad3C are localized in the nuclei of mature hepatocytes. (B) JNK activation results in phosphorylation of both Smad2L and Smad3L. pSmad3L can move to the nucleus even when the C-terminal is not phosphorylated. On the other hand, pSmad2L translocate to the nucleus only after their COOH-tail is phosphorylated by TβRI. pSmad3L, pSmad3L/C and pSmad2L/C are observed in the nuclei of MFB and pre-neoplastic hepatocytes.

In canonical Smad signaling pathway, the activated TβRI is well established as being starting point for signal propagation to Smad3 (33). After Smad3 is phosphorylated by the activated TβR1 on the C-terminal SXS motif (Fig. 1A), pSmad3C forms the complex with the common partner Smad4 (11). The complex translocates to the nucleus, where they regulate target gene expression both direct DNA binding and by interaction with other transcription factors, co-activators, and co-repressors (34). This pathway is regulated by several auto-inhibitory feedback loops (35). In particular, Smad7 interacts stably with activated TβR1 receptor to inhibit TGF-β-mediated COOH tail phosphorylation of Smad2 and Smad3 (36,37).

TGF-β represents a major growth inhibitory signal in normal epithelial cells such as hepatocytes (6). In the context of cell cycle control, the most important targets of action by TGF-β are the genes encoding the two CDK inhibitors p15^INK4B^ and p21^Cip1^, that inhibit CDKs and downregulate c-Myc expression (38). As the cytostatic effects of TGF-β are reversible, pSmad3C is negatively regulated by a number of phosphatases, such as protein phosphatase 1A, magnesium-dependent (PPM1A) (39). PPM1A overexpression abolishes, and PPM1A depletion enhances TGF-β-induced anti-proliferative responses (39). Furthermore, PPM1A binds with pSmad3C to facilitate nuclear export of dephosphorylated Smad3 to the cytosol (22). Thus, C-tail dephosphorylation mediates Smad recycling for further signaling and thereby links duration of signaling to the presence of activated TβRI (40).

### Non-canonical Smad signaling pathways

#### Mitogenic JNK/pSmad3L signaling

JNK is a serine/threonine kinase affecting proliferation, differentiation, survival, and migration. JNK can phosphorylate Smad3 at linker region (26). In contrast to cytoplasmic retention of pSmad2L, pSmad3L is not retained in the cytoplasm (Fig. 1B). Both pSmad3C and pSmad3L can form hetero-complexes with Smad4, and Smad complex move to the nucleus (19). Because nuclear hetero-oligomerization is essential to assembly of target-specific transcriptional complexes (34), Smad3 can utilize 2 different phospho-domains to transmit different signals, as both a tumor suppressor and a tumor promoter (23).

JNK-mediated pSmad3L and TβRI-mediated pSmad3C signals oppose each other; most importantly, the balance can shift between cell growth and growth inhibition. Linker phosphorylation of Smad3 indirectly inhibits its COOH-terminal phosphorylation and subsequently suppresses tumor-suppressive pSmad3C signaling. Linker phosphorylation can modify COOH-terminally phosphorylated Smad2/3 signaling (13–15,19,20,25). By using genetic as well as pharmacologic approaches, we showed that blockade of linker phosphorylation abolished oncogenic properties in Ras-transformed cells and restored the TβRI/pSmad3Cmediated tumor-suppressive function present in parental epithelial cells (25).

#### Invasive/fibrogenic TβRI/JNK/pSmad2L/C signaling

Activated JNK retains most Smad2 proteins in the cytoplasm (13,25). Smad2 can accumulate in the nucleus only if its C-terminus is phosphorylated under conditions of sustained linker phosphorylation by JNK (Fig. 1B). Smad2 or Smad3 deficient mouse embryo-derived fibroblasts suggest that both Smad2 and Smad3 are required for induction of plasminogen activator inhibitor (PAI)-1 (41). Smad3 cooperates with Smad4 to activate the PAI-1 promoter in a TGF-β independent manner (42). Smad3 mutant (Smad3SD), in which the C-terminal serines are replaced by aspartic acids, localized in the nucleus to activate PAI-1 transcription in TGF-β independent fashion (43). Importantly, Smad3SD mutant lacks induction of target genes required for growth inhibition (43). Moreover, Smad3 phospho-mimetic mutation in the linker domain enhance PAI-1 mRNA and protein (44). pSmad2L/C undergoes translocation to the nucleus, where it binds to pSmad3L and Smad4 complex (18,15), which in turn stimulates PAI-1 transcription (18). PAI-1 facilitates cell invasion (45) and induces ECM deposition (46).

#### Mitogenic TβRI/CDK/pSmad2/3L/C signaling

**Figure 2:**
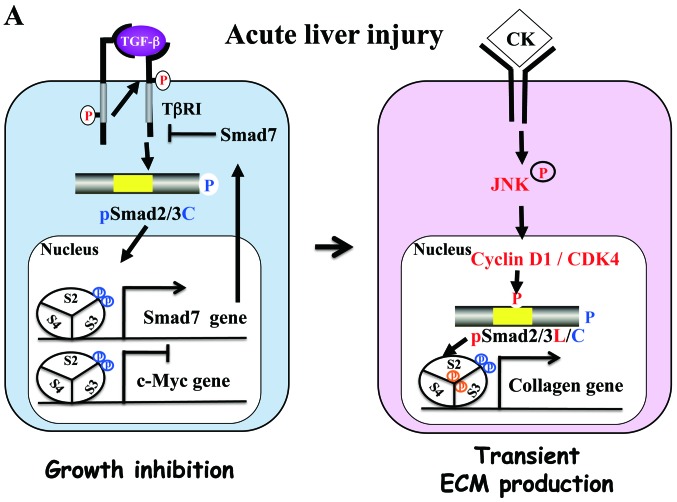

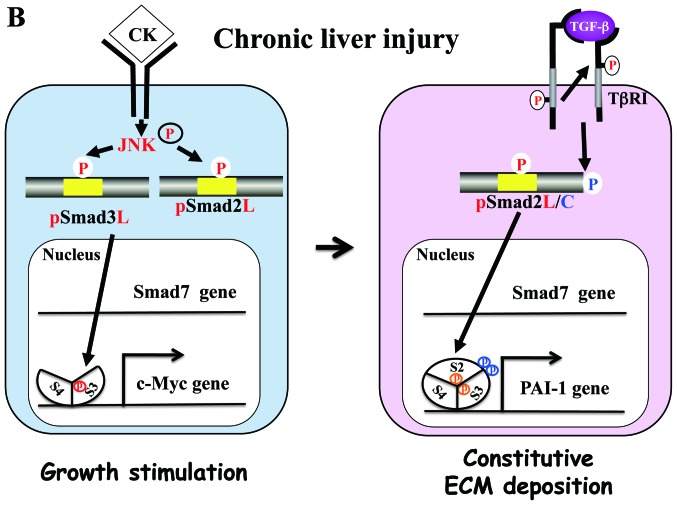
Differential regulation of TGF-β/Smad signaling after acute or chronic liver injuries. (A) After acute liver injury, loss of hepatocytes rapidly induces a wave of cell proliferation. TGF-β plays important roles during liver regeneration. TGF-β inhibits HSC growth by downregulating c-Myc expression by pSmad2C and pSmad3C pathways (left); TGF-β signaling in turn enhances HSC growth and collagen synthesis via the CDK4-dependent pSmad2L/C and pSmad3L/C pathways induced by cytokine (CK) signal (right). However, Smad7 induced by pSmad3L/C signal terminates the fibrogenic phospho-Smad signaling. This negative-feedback mechanism of the fibrogenic TGF-β/CK signal results in a transient collagen synthesis in the activated HSC, which may thus contribute to tissue repair. (B) Several conditions in chronically damaged livers favor human hepatocarcinogenesis, mostly resulting from recurrent cycles of cellular proliferation, inflammation and fibrosis. In MFB and pre-neoplastic hepatoycytes, CK activates JNK, which phosphorylates Smad2L and Smad3L (left). The JNK-mediated Smad3L phosphorylation leads to a hetero-complex of Smad3 with Smad4 in the nucleus where the complex stimulates MFB and pre-neoplastic hepatycyte growth by upregulation of c-Myc transcription. After COOH-tail phophorylation of cytoplasmic pSmad2L by TGF-β signal, pSmad2L/C translocates to the nucleus where it binds to the pSmad3L and Smad4 complex, which then stimulates plasminogen activator inhibitor type I (PAI-1) gene transcription (right). In contrast of Smad7 induction in HSC via pSmad3C pathway, pSmad3L cannot induce Smad7 in MFB and pre-neoplastic hepatoycyte (left). Under a low level of Smad7, the fibrogenic phospho-Smad signaling can constitutively promote ECM deposition by MFB, which may eventually develop into accelerated liver fibro-carcinogenesis.

Liu group previously reported that Smad3 was phosphorylated by CDK4 *in vivo* and *in vitro* (47). CDK4-mediated phosphorylation of Smad3 at its linker region inhibits its transcriptional activity and the anti-proliferative activity of TGF-β in fibroblasts (14,48). We have confirmed that the nuclear cyclin D1/CDK4 complex of fibroblasts activated by TGF-β and PDGF signaling directly phosphorylates the linker segment of pSmad2C to produce pSmad2L/C (15). The expression of c-Myc in fibroblasts is initially repressed by TGF-β, but subsequent cyclin D1/CDK4 undergoes a complete functional change to stimulate c-Myc (15). TGF-β inhibits cell growth by downregulating the c-Myc via the pSmad2C and pSmad3C pathways (Fig. 2A, left). Moreover, Hayashida *et al* reported that pSmad3L/C increases collagen I synthesis in human mesangial cells (49) (Fig. 2A, right).

### Non-Smad pathway

TGF-β also uses non-Smad signaling pathways including JNK and p38 MAPK pathways to convey the same invasive/ fibrogenic signals (50). Tumor necrosis factor (TNF)-receptor-associated factor 6 (TRAF6) and TGF-β associated kinase 1 (TAK1) have recently been shown to be crucial for the activation of the MAPK (51–53). TAK1 pathway is known to regulate cell survival, migration and invasion.

Especially important among genes induced by JNK pathway are the 2 immediate early genes encoding the Fos and c-Jun transcription factors. Once synthesized, these proteins can associate with one another to form activator protein (AP)-1, a widely acting heterodimeric transcription factor that is often found in hepatocarcinogenesis and liver fibrosis (54). TGF-β and pro-inflammatory cytokines elicit signaling responses through JNK/non-Smad pathway (50). In JNK1^−/−^ mice, both fibrosis and HCC development are prevented. Collagen deposition is marked in wild-type and JNK2^−/−^ mice, but is less dense in JNK1^−/−^ mice, suggesting the importance of JNK1 in development of liver fibrosis (55). JNK1^−/−^ mice exhibit impaired liver carcinogenesis, with smaller and fewer tumor masses (56). Importantly, JNK1^−/−^ mice displayed decreased HCC proliferation in a carcinogenic model and decreased hepatocytic growth in a model of liver regeneration. In both instances, impaired proliferation is caused by increased expression of p21^WAF1^, a cell-cycle inhibitor, and reduced expression of c-Myc, a negative regulator of p21^WAF1^.

These observations suggest cross-talk between TGF-β induced non-Smad signaling and non-canonical Smad pathway in the nucleus appear to play an important role during the liver fibrosis and carcinogenesis. The recognition of non- Smad and non-canonical Smad pathway as a potent driver of fibrocarcinogenesis makes it urgent to investigate in more detail the molecular mechanisms by which TGFβ promotes its oncogenic effects.

## 3. Differential regulation of the Smad phospho-isoform signaling between acute and chronic liver diseases

### Acute liver injury

Compensatory growth of the liver to regain mass lost by partial hepatectomy and chemical damage is orchestrated by interplay of positive and negative polypeptide cytokines and growth factors (57). After acute liver damage, transient release of inflammatory cytokines participate in the restoration (57). Patches of quiescent cells are stimulated by cytokines to move into a primed state (G0→G1), when growth factors can stimulate DNA synthesis and cellular replication (58). If hepatocytes are damaged so that this response is impaired, hepatocytes may be derived from progenitor/stem cells located in the vicinity of the canals of the Hering (58).

We reported that plasma TGF-β levels increased after acute liver injury, and the anti-proliferative response to TGF-β decreased in hepatocytes by downregulation of TGF-β receptor expression in rat livers (3,4). In HSC, whenever TGF-β is increased, TGF-β could transduce its signal for ECM production via its receptor because signaling receptors were expressed constantly (4). From the available evidence, examples of acute liver hepatitis are followed by complete or near-completed resolution and return of the liver to normal (58).

We further examined in more detail TGF-β signaling in hepatocytes and HSC during acute liver injury, focusing on pSmad2L/C and pSmad3L/C pathways in chemically injured rat livers (18,26). These phospho-isoforms are involved in collagen synthesis and transmit a proliferative, invasive TGF-β signal in mesenchymal cells (18,26). Nuclear localization of pSmad2L/C and pSmad3L/C is seen in the activated HSC (26). In particular, strong Smad2/3 phosphorylation at the COOH-tail and threonine residues in the linker regions is observed in the activated HSC (unpublished data). Because TGF-β, pro-inflammatory cytokines and PDGF activate JNK pathway in HSC (26), pro-inflammatory cytokines and PDGF can convert a cytostatic TGF-β signal to a collagen-producing character in activated HSC under the influence of inflammatory microenvironments (Fig. 2A, right). Collectively, pSmad2L/C and pSmad3L/C signaling may mobilize HSC from the space of Disse to sites of damage, where the activated HSC contribute to tissue repair by producing large amounts of collagens.

In HSC after acute liver injury, TβRI activated by endogenous TGF-β signal phosphorylated Smad3C, further upregulating Smad7 transcription (Fig. 2A, right) (59). Subsequently, Smad7 terminates fibrogenic signals mediated by pSmad2L/C and pSmad3L/C, and could be involved in transient response to the autocrine TGF-β signal after acute liver injury (26,59). In the same way, the activation of Smad2/3 was tightly restricted in primary cultured HSC (26,59). Taken together, Smad7 is involved in this tight restriction of the non-canonical Smad signaling in HSC and regulates the intensity and duration of the TGF-β responses (60).

### Chronic liver injury

Chronic inflammation causes progressive liver fibrosis (Fig. 2B). Fibrogenesis is a mechanism of wound healing and repair (61). However, prolonged injury causes deregulation of the normal processes and results in extensive deposition of ECM proteins and fibrosis (62). Activation of HSC is a key step in liver fibrogenesis (62). When freshly isolated and cultured, quiescent HSC have a low proliferative rate and very modest fibrogenic potential and lack of contractile properties (63). Therefore, the main function of these quiescent HSC is considered to be the storage and metabolism of vitamin A (2). However, following liver injury of any etiology, HSC undergo activation. Activated HSC show increased proliferation, motility and ECM production (64,65). A number of cytokines, continuously released by damaged Kupffer cells and endothelial cells, can change activated HSC to myofibroblasts (MFB) (66). These include TGF-β, PDGF and ET-1, which stimulate transcription factors such as Sp1, c-jun, STAT-1 and Smad proteins that regulate gene expression (67–70). MFB perpetuate their own activation through several autocrine loops, including the secretion of TGF-β and upregulation of its receptors (59). Following chronic liver injury, there is a marked accumulation of α-smooth muscle actin (α-SMA) positive cells at the sites of active liver fibrosis (71,72). The most powerful growth factor for MFB is PDGF (68). Moreover, following cell activation, there is upregulation of PDGF receptors in MFB, which in turn can secrete this potent mitogen (73).

During transdifferentiation from HSC to MFB in culture, pSmad3C-mediated signal decreases while pSmad3L pathway predominates (18). The observations fully support the finding of pSmad3L rather than pSmad3C in nuclei of α-SMA-immunoreactive MFB in portal tracts of chronically HCV-infected liver specimens (27). The presence of α-SMA is associated with transdifferentiation of HSC into scar-forming MFB, an event that is considered pivotal in the fibrogenic response (2).

In contrast to a transient increase in Smad7 in the activated HSC after acute liver injury, Smad7 remains at a low level in MFB throughout chronic liver injury (59). Because Smad7 cannot be induced by the pSmad3L pathway (unpublished data), the lack of Smad7 induction in MFB during chronic liver disease might lead to constitutive fibrogenic TGF-β (59,74,75). Accordingly, Smad7 overexpression results in less accumulation of interstitial collagens and improves liver fibrosis (76). Moreover, interferon (IFN)-γ displays antifibrotic effects by upregulation of Smad7 expression (77).

Although MFB have been considered the primary cells involved in development of liver fibrosis, possible direct involvement of hepatocytes in fibrosis has not been examined. In parallel with emergence of epithelial-to-mesenchymal (EMT) paradigm in fibro-carcinogenesis, a large body of work has established roles for epithelial cells as important mediators of progressive fibrosis (27,62). During progression of HCV-related chronic liver disorders, our current data indicated that hepatocytes affected by chronic inflammation undergo transition from the tumor-suppressive pSmad3C pathway, which is characteristic of mature hepatocytes, to the JNK/pSmad3L pathway, which appears to favor the state of flux shown by MFB, accelerating liver fibrosis. These phenomena were also observed in HBV-related liver disease (28).

### Hepatocarcinognesis

HCC is the sixth most common cancer and third most frequent cause of cancer-related death worldwide (78,79). Although there is a growing understanding of the molecular mechanisms that induce hepatocarcinogenesis, the mechanisms have not been completely elucidated. Chronic infections with HBV or HCV appear to be the most significant causes of HCC (80). Recent studies reveal that the development and progression of HCC are caused by the accumulation of genetic changes, thus resulting in altered expression of cancer-related genes (81).

As HBV contains partially double stranded-DNA, it can directly cause HCC by integrating its DNA into the host genome. HBV genomic integration is present in 85 to 90% of livers developing HBV-related HCC, usually even before the development of HCC (82). Integration of HBV DNA is not restricted to HCC, but is also found in non-tumor tissue in patients with chronic HBV infection (83,84). HBV integration induces a wide range of genetic alterations within the host genome, including chromosomal deletions, translocations, production of fusion transcripts, amplification of cellular DNA and generalized genomic instability (85,86). HBx protein encoded by the X gene has been suspected as a viral oncoprotein participating in hepatocarcinogenesis (87). HBx was shown to potentiate c-Myc-induced liver carcinogenesis in transgenic mice (88).

Unlike HBV, HCV is a positive, single-strand RNA virus, apparently incapable of integration into the host’s genome. The HCV components modulate a number of cellular regulatory functions by targeting a wide spectrum of cellular signaling pathways (89,90–96). HCV core expression has been shown to induce activation of the JNK pathway in regulation of vascular endothelial growth factor (96). NS5A acts as a positive regulator of the JNK signaling pathway by interacting with TNF receptor-associated factor 2, which may be highly important in HCV pathogenesis (97). In an HCV infection model, Lin *et al* demonstrated that HCV directly induced TGF-β release from hepatocytes in a reactive oxygen species (ROS)-dependent and JNK-dependent manner (98). Moreover, recent studies using transgenic mouse models indicated that HCV is involved directly in hepatocarcinogenesis. Three different HCV core transgenic lines develop liver steatosis and HCC (99–101).

**Figure 3:**
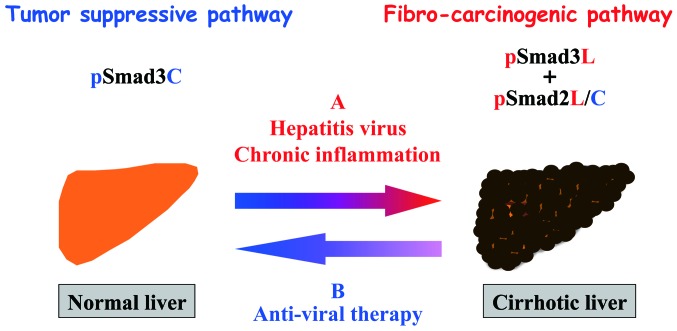
Reversible Smad phospho-isoform signaling between tumor suppression and fibro-carcinogenesis. (A) As human hepatitis virus-related chronic liver diseases progress, chronic inflammation and hepatitis virus additively shift hepatocytic Smad phospho-isoform signaling from tumor suppressive pSmad3C to carcinogenic pSmad3L and fibrogenic pSmad2L/C pathways, accelerating liver fibrosis and increasing the risk of HCC. (B) Effective anti-viral therapy can reverse Smad phospho-isoform signaling from fibro-carcinogenesis to tumor suppression.

We have shown that in patients with chronic liver disease progression, HBV or HCV components and pro-inflammatory cytokine additively activate JNK to shift Smad phospho-isoform signaling from tumor-suppressive TβRI/pSmad3C pathway to carcinogenic JNK/pSmad3L pathway and fibrogenic pSmad2L/C pathway, accelerating liver fibrosis and increasing the risk of HCC (Fig. 3A). To support this notion, high level of linker Smad3 phosphorylation is reported both in HCC specimens and human HCC cell lines (102). Moreover, specimens from patients with chronic hepatitis B who develop HCC show abundant hepatocytic Smad3L but limited Smad3C phosphorylation in hepatocytic nuclei, whereas other patients with abundant heptocytic pSmad3C but limited pSmad3L do not develop HCC (28). The same relationships are observed in human hepatitis C virus-related hepatocarcinogenesis (27).

## 4. Reversible Smad signaling after successful therapies against hepatitis viruses

Chronic hepatitis B and C are now treatable diseases. Interferon therapy and nucleoside analogues are available for HBV. Lamivudine and four other nucleoside and nucleoside analogues have been licensed (103): adefovir (in 2002) (104), entecavir (in 2005) (105), telbivudine (in 2006) (106), and most recently, tenofovir disoproxil fumarate (in 2008). These nucleoside analogues suppress HBV replication through inhibition of reverse transcriptase and DNA polymerase, and inhibit reverse transcription of pregenomic RNA to HBV DNA (103). On the other hand, the current treatment of hepatitis C is pegylated IFN (PEG-IFN)-α, given by subcutaneous injection once weekly, and oral ribavirin (RBV) daily. RBV is a guanosine nucleoside analogue. This agent shows only modest activity against hepatitis C but it increases the activity of IFN-α when the 2 agents are used in combination (107). Efficacy of PEG-IFN and RBV has been investigated in several controlled trials that demonstrated an overall SVR rate of 40 to 50% (107). However, limitations of IFN and RBV treatment have prompted a continuing search for improved therapies. Various molecular targets are the focus of anti- HCV drug development, several new NS3 protease inhibitors, NS5b nucleoside polymerase inhibitors, and non-nucleoside polymerase inhibitors are being assessed in phase III studies.

Previous studies have shown that successful anti-viral therapy can improve biochemical liver function parameters as well as histological findings (108). Patients with mild liver fibrosis are likely to show histologically evident decreases in fibrosis and inflammation after a sustained virological response (SVR) in response to IFN treatment against HCV infection (108). Furthermore, treated patients show marked reduction in decompensated liver disease and HCC occurrence (109,110). Patients with advanced fibrosis, however, retain relatively low but still considerable risks of HCC occurrence and hepatic decompensation despite having attained SVR (109).

Clinical analyses of pSmad3L and pSmad3C in liver disease progression have provided substantial mechanistic insight. After achievement of SVR, IFN or an oral nucleoside therapy could restore Smad phospho-isoform signaling from oncogenic pSmad3L to tumor-suppresssive pSmad3C pathway shown by normal hepatocytes both in chronic hepatitis B and C (Fig. 3B) (31,32). In contrast, patients with advanced liver fibrosis progressed to HCC despite improved inflammatory activity, because hepatocytes maintained high pSmad3L and low pSmad3C signaling (31). One reason why pSmad3L level remains high may be that chronic HBV or HCV infection and chronic inflammation no longer play critical roles in HCC development in later cirrhotic livers after pre-neoplastic hepatocytes have acquired oncogenic signaling caused by genetic alteration and epigenetic changes. These clinical observations support roles for pSmad3C as a tumor suppressor and pSmad3L as a promoter during hepatic carcinogenesis.

Antiviral therapy can achieve recovery of liver inflammation and fibrosis in HBV or HCV infected patients. Moreover, we also demonstrated that hepatocytic tumor-suppressive pSmad3C signaling shifted to fibro-carcinogenic pSmad3L signaling as the livers progressed from chronic hepatitis B and C infection to HCC, and suppression of liver inflammation and regression of fibrosis by an anti-viral therapy resulted in the reduction of pSmad3L and increase in pSmad3C signaling. Likewise, oncogenic c-Myc and fibrogenic PAI-1 expression was significantly decreased in the livers post-anti-HBV or HCV-treated patients. Both HBV and HCV trigger changes in gene expression, which is mediated by genetic or epigenetic alterations. The contribution of HBV to HCC involves the expression of HBx; for HCV, the core protein, and nonstructural protein NS3 and NS5A contribute to oncogenic transformation (94,97). Suzuki *et al* reported a −0.6 improvement in HBe-antigen negative chronic hepatitis B patients after one year lamivudine treatment (111). We also found that fibrosis regressed −1 point after 52 weeks of treatment of anti- HBV treatment in the livers of chronic HBV patients (32). On the contrary, fibrosis regression rate was −0.28 point/year in HCV infected patients after SVR (31). These results indicate that treatment with nucleoside analogues resulted in a 3–4 times faster fibrosis regression rate in HBV-infected patient livers compared with that of IFN treated HCV-infected patient. Notably, the fibro-carcinogenesis regression rate was much faster in HBV than that of HCV-related liver disease (32). These data coincide with the clinical observations that decompensated HBV-infected patients often show disappearance of ascites or jaundice even in advanced stages after an oral nucleoside therapies. As the hepatic fibrocarcinogenesis seems to be a multistep process, the differences of virus specific genetic changes and biological consequences may cause the alternation of fibrosis regression rate between HBV and HCV. During HCV-related carcinogenesis, JNK-activated chronic inflammation confers a selective advantage on preneoplastic hepatocytes by shifting Smad3 signaling from the tumor-suppressive pSmad3C to the oncogenic pSmad3L pathway (27). On the other hand, HBx oncoprotein participates directly in hepatocarcinogenesis by shifting hepatocytic Smad3-mediated signaling from tumor suppression to oncogenesis in patients with chronic hepatitis B infection (28). Such differences may result in faster progression or regression rate of fibrocarcinogenesis in HBV patients compared with that in HCV patients during, before, and after anti-viral therapy.

## 5. Problems and future perspectives

In this review, we describe TGF-β/Smad phospho-isoform signaling during acute and chronic liver diseases. Anti-viral therapy has been shown to decrease the risk of HCC by shifting fibro-carcinogenic pSmad3L signaling to tumor-suppressive pSmad3C in hepatocytes. Therefore, understanding molecular mechanisms of human fibro-carcinogenesis is of fundamental importance in guiding development of effective prevention and treatment for hepatic fibrosis and HCC. Additionally, Smad phospho-isoform signaling can be used as a new predictive biomarker for early assessment of pharmacologic interventions to suppress human fibro-carcinogenesis.
